# Mendelian Randomization Suggests a Causal Link Between Glycemic Traits and Thoracic Aortic Structures and Diseases

**DOI:** 10.1016/j.jacbts.2026.101517

**Published:** 2026-03-23

**Authors:** Nimrat Grewal, John Elefteriades

**Affiliations:** a Amsterdam University Medical Center, Location AMC, Amsterdam, the Netherlands; b Aortic Institute at Yale-New Haven Hospital, Yale University School of Medicine, New Haven, Connecticut, USA

Daria et al^1^ present an ambitious and technically sophisticated analysis to explore whether glycemic traits influence thoracic aortic structure and risk of thoracic aortic aneurysm or dissection (TAAD). The breadth of methods, careful sensitivity analyses, and focus on an epidemiologically intriguing question are commendable. The relationship between glycemic traits and thoracic aortic disease has long been marked by inconsistent and counterintuitive observations,^2^^,^^3^ and the authors take an important step in probing this complexity.

However, interpreting the results as evidence for a causal relationship between glycemic traits and thoracic aortopathy requires caution, not because of limitations in statistical rigor, but because of a fundamental mismatch in phenotypes across data sets.

Although the authors frame their findings in the context of aortopathy and aneurysm risk, the imaging-derived phenotypes originate from the UK Biobank (UKBB) imaging cohort, which predominantly reflects normal anatomical variation.^4^ Their reported mean “maximum ascending aortic area” (∼853 mm^2^), corresponding to an approximate diameter of ∼33 mm, lies squarely within the physiological range. Importantly, neither the paper nor the Supplemental Appendix reports minimum, median, or distributional measures of aortic size, underscoring that the data set does not capture pathological dilation or early aortopathic remodeling. Consequently, the genetic signals identified in these analyses map to determinants of normal vessel structure rather than mechanisms of aneurysm formation.

The authors appropriately use the Million Veteran Program for magnetic resonance (MR) outcome analyses, providing >7,000 TAAD cases. Yet, this juxtaposes physiological UKBB imaging traits with overt disease phenotypes from the Million Veteran Program. Such cross-cohort exposure–outcome MR designs are common, but in this context, the biological continuum between the 2 phenotypes is not well established. Furthermore, UKBB epidemiological analyses rely on broad International Classification of Diseases-based TAAD definitions that aggregate type A and B dissections as well as ruptured and nonruptured aneurysms, each with distinct pathophysiology and clinical trajectories.

Daria et al^1^ highlight glycemic pathways that may influence vascular elasticity or baseline aortic wall behavior, and the multiomic strategy offers valuable avenues for hypothesis generation. But, these results should not be interpreted as confirming a causal influence of glycemic traits on thoracic aortopathy. The analyses address physiological vessel variation; whether these pathways also modulate aneurysm progression remains unresolved. It is further worth noting that some of the exposure definitions and measurements reflect the practical constraints of large biobank data sets. In UKBB, glucose levels are in part based on random rather than fasting measurements, and blood pressure values are adjusted for antihypertensive treatment using standard correction factors. Although these approaches are widely accepted, they inevitably introduce variability when studying subtle vascular phenotypes such as aortic diameter, strain, and distensibility. In addition, MR imaging-based measurements of the ascending aorta may not uniformly capture distinct anatomical regions, particularly the aortic root vs the tubular segment. These aspects do not undermine the overall quality of the work, but they further support a cautious interpretation when extending population-based aortic traits to disease-level thoracic aortopathy.

Future work should integrate this analytic framework with cohorts enriched for true thoracic aortic disease, including anatomically resolved outcomes and longitudinal imaging. Combining genomic variation with aneurysm tissue analyses, single-cell and single-nucleus sequencing, proteomics, and epigenomics will be essential for determining whether glycemic-associated pathways influence matrix remodeling, aortic growth, or dissection risk.

This study is innovative and methodologically rigorous, and it raises valuable hypotheses regarding metabolic influences on vascular biology. However, distinctions between physiological aortic structure and pathological aortopathy must remain central when interpreting causality. The work provides a strong foundation for deeper mechanistic investigation, but it is not yet definitive regarding glycemic influence in thoracic aortic disease.
